# Dr. Manuel Martinez-Maldonado: A Pioneer in Renal Therapeutics and Mentor to a Generation of Minority Physician-Scientists

**DOI:** 10.7759/cureus.68776

**Published:** 2024-09-06

**Authors:** Jorge R Figueroa-Quintana, Simran Rajput, David J Chow, Estela S Estapé

**Affiliations:** 1 Medicine, San Juan Bautista School of Medicine, Caguas, PRI; 2 Research, San Juan Bautista School of Medicine, Caguas, PRI; 3 Research, Medical Sciences Campus, University of Puerto Rico, San Juan, PRI

**Keywords:** minority representation in medicine, medical education, minority health, academic leadership, mentoring, renal physiology, nephrology, renal therapeutics, historical vignette, biography

## Abstract

Dr. Manuel Martinez-Maldonado is a distinguished Puerto Rican internist, nephrologist, physician-scientist, mentor, and prolific writer whose leadership in academic and clinical settings has significantly advanced the fields of nephrology, renal physiology and pharmacology, fluids and electrolyte metabolism, calcium metabolism, hypertension research, and medical education. His research on electrolyte imbalances has led to innovative hypercalcemia treatments, notably furosemide with IV fluid therapy. This is an approach that, combined with pharmacotherapy using calcitonin and bisphosphonates, became the standard practice for managing hypercalcemia until specific therapies became available. His nephrology research team and laboratory in the San Juan VA (Veterans Affairs) Medical Center and the Medical School of the University of Puerto Rico were internationally renowned. Throughout his career, he fostered a culture of mentorship while spearheading superb clinical teaching and research initiatives. His transformative tenures at several institutions, including Baylor College of Medicine; the University of Puerto Rico-Medical Sciences Campus; the VA medical centers in Atlanta, Houston, and San Juan; Emory University; Oregon Health Sciences University; Ponce School of Medicine; and the University of Louisville School of Medicine demonstrate his lasting contributions to medical science and education. His interdisciplinary approach, advocacy for kidney and clinical research, and contributions to understanding the renin-angiotensin system and the role of sodium-potassium-activated adenosine triphosphatase in renal concentration mechanisms illustrate his enduring impact on renal physiology and human health.

## Introduction and background

Medical pioneer and mentor are two qualities that underscore the impact of Dr. Manuel Martinez-Maldonado's life and choices on future generations. The definition of a pioneer depends on the perspective of the one crafting it. However, what typically comes to mind is someone who dares to take innovative actions that conjure new frontiers and discovery or being the first recognized for achievements in specific areas. When we refer to a medical pioneer, we think of someone whose achievements have significantly advanced our understanding of health and improved overall well-being. While this medical pioneer can also make time to be a dedicated researcher and mentor, one finds a person who motivates others to excel, such as Dr. Martinez-Maldonado.

**Figure 1:**
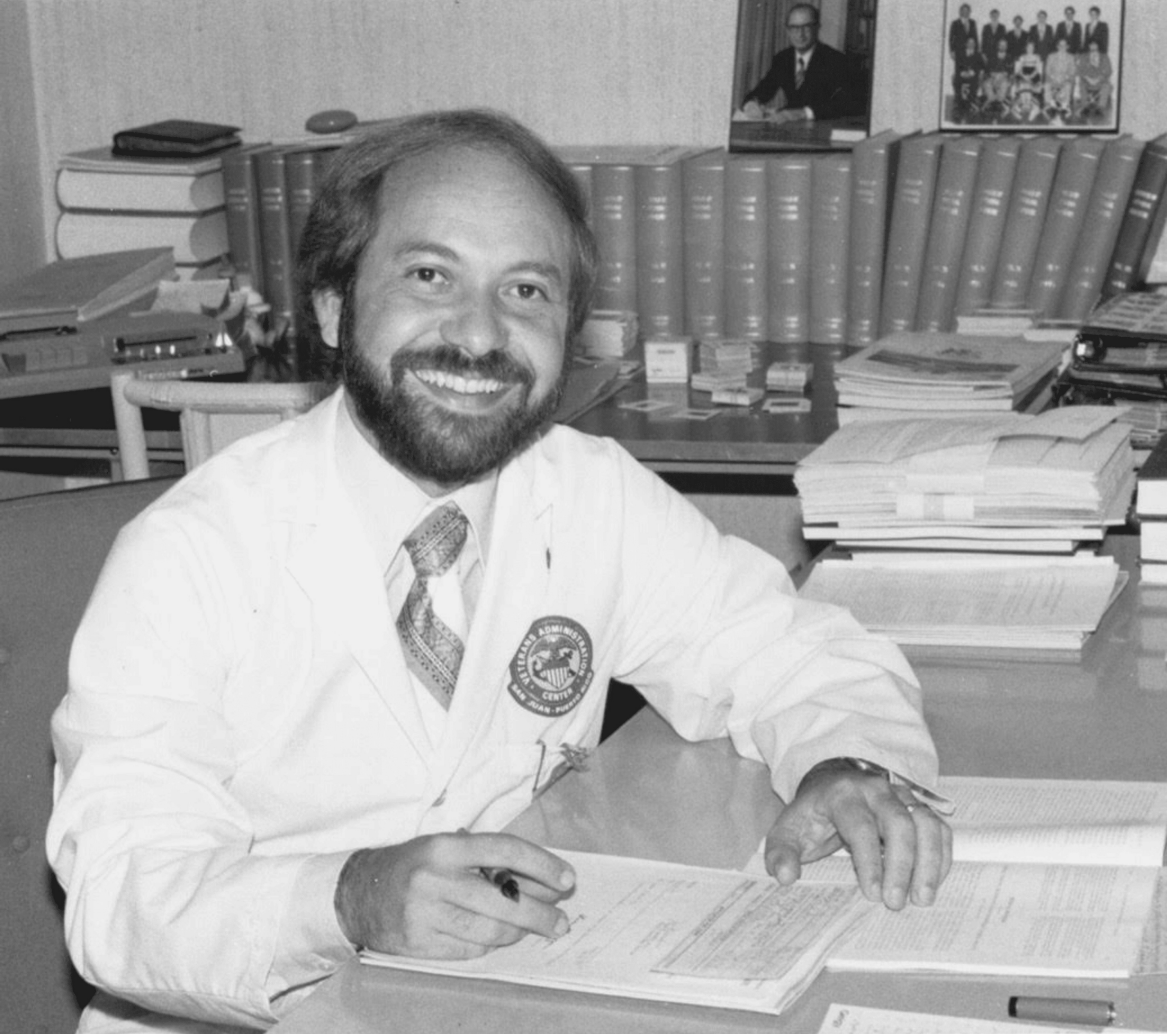
Dr. Manuel Martinez-Maldonado. Dr. Manuel Martinez-Maldonado approved the publication of this photograph.

Dr. Martinez-Maldonado's leadership and mentorship have profoundly shaped the careers of dozens of scientists, physicians, and clinical researchers. His curiosity, resilience, and passion for his work guided his remarkable career and set the expectations of those training and working alongside him. His keen observation, listening skills, and trademark ability to interject mid-conversation with valuable insights helped make discussions richer intellectually and more focused clinically. His mentees often highlight his guidance as instrumental in their professional development and success. The impact of Dr. Martinez-Maldonado's career as a physician and mentor has helped enhance patient care and drive clinical research forward. Recognizing his achievements and contributions, he became the first underrepresented minority inducted into the American Society for Clinical Investigation [1] and the first Puerto Rican elected to the Institute of Medicine, now the National Academy of Medicine. He was also one of the few Puerto Rican members of highly recognized national and international medical and scientific associations, including the Association of American Physicians, the American Academy of Arts and Sciences, and a Fellow of the American Society for the Advancement of Science (Figure 1).

## Review

Early life and career as a physician-scientist 

Born in 1937 in the small town of Yauco, Puerto Rico, Dr. Martinez-Maldonado was the only child in a family of avid readers. His mother, a history and English teacher, instilled in him a love of reading, while his uncle, an entomologist, imparted his love for science. Torn between his passion for literature and his drive to become a physician, he began his formal education at the University of Puerto Rico, earning a bachelor's degree in chemistry and demonstrating his talent in poetry, tacitly approved by his mentor, Juan Ramon Jimenez, 1956 Nobel Prize in Literature. He went to Philadelphia to pursue a medical career, earning an M.D. degree in 1961 from Temple University School of Medicine (now the Lewis Katz School of Medicine at Temple University).

Dr. Martinez-Maldonado completed his rotating medical internship at St. Charles Hospital in Toledo, Ohio. Following his internship, he returned to Puerto Rico in 1962 to complete internal medicine residency training at the San Juan VA (Veterans Affairs) Medical Center and the University of Puerto Rico School of Medicine. There, he was appointed chief resident of internal medicine in 1964, presaging Dr. Martinez-Maldonado's long career not only as an exceptional and innovative physician but also as a leader in his field, an advocate for his colleagues and his patients, and a mentor and guide to hundreds of medical students, resident physicians, and fellows.

**Figure 2:**
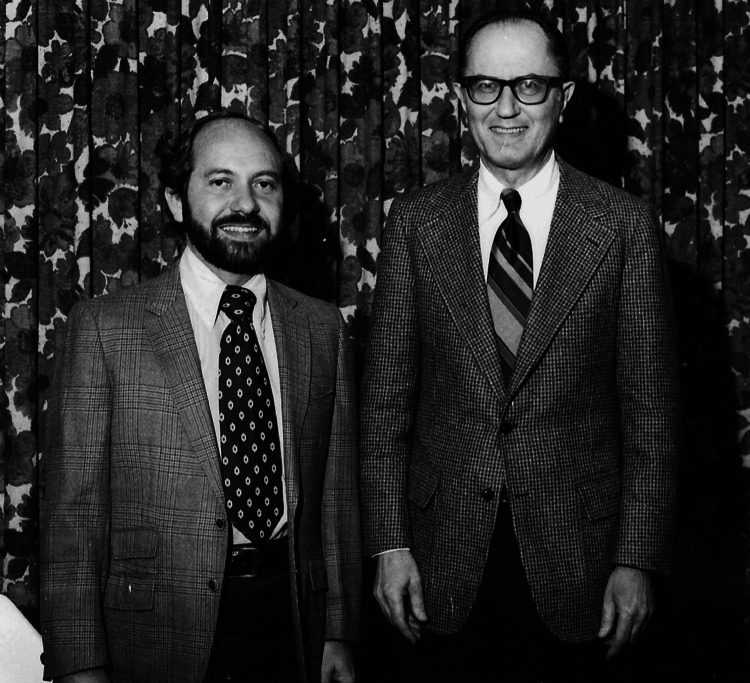
Dr. Manuel Martinez-Maldonado, with one of his primary mentors in nephrology, Dr. Donald W. Seldin. Dr. Martinez-Maldonado approved the publication of this photograph.

Following his internal medicine residency, Dr. Martinez-Maldonado pursued postdoctoral fellowship training in nephrology, which he completed in 1967 at the University of Texas Southwestern (UTSW) Medical Center in Dallas, Texas. At UTSW, he trained under two eminent internists, academicians, and renal physiologist-nephrologists Dr. Donald W. Seldin [2] and Dr. Floyd C. Rector, Jr. [3]. Dr. Martinez-Maldonado credits them with transforming his ability to think and approach clinical problems and how to react, present, and analyze them (Figure 2).

After completing his fellowship training, UTSW hired Dr. Martinez-Maldonado as an instructor of medicine and medical director of the Chronic Dialysis unit of Parkland Memorial Hospital in Dallas. Later, the acclaimed surgeon Dr. Michael DeBakey recruited him to Baylor College of Medicine. At Baylor, Dr. Martinez-Maldonado assumed not only the associate directorship of the Renal Section but also joined as the Chief of the Nephrology Section at Houston VA Medical Center. Dr. Martinez-Maldonado joined two other UTSW alumni who had been his colleagues in training and were recent Baylor recruits, Dr. Wadi Suki, at Houston Methodist and Dr. Garabed Eknoyan, at Ben Taub Hospital, Houston to form a nephrology department regarded as one of the strongest in the country. Between 1969 and 1973, Dr. Suki, Dr. Martinez-Maldonado, and Dr. Garabed Eknoyan published over 30 original peer-reviewed articles on clinical nephrology, renal physiology, and pathophysiology.

Contributions to human health

Dr. Martinez-Maldonado is an esteemed physician and scientist known for nephrology, hypertension research, and medical education. He has significantly improved our understanding of kidney function and blood pressure regulation [4,5]. He has also trained and impacted young physician-scientists to develop niche research labs. With 127 original articles in peer-reviewed journals, including his identification and description of the possible role of Na,K-ATPase in the renal concentrating mechanism in science [6], Dr. Martinez-Maldonado has firmly established himself as an innovator and leader in nephrology.

In a study published in the *New England Journal of Medicine*, Dr. Martinez-Maldonado and his co-authors introduced a treatment protocol for patients with acute hypercalcemia [7]. This protocol utilized saline infusion and furosemide therapy to stimulate urinary excretion of calcium, thereby reducing excess plasma calcium levels by causing hypercalciuria. At the time, it served as the only treatment option for such clinical presentations and quickly became the standard of care. He co-authored another study in the *New England Journal of Medicine* based on combination treatments for patients with excessive ascites [8]. The study found that combining ascitic fluid and furosemide infusion enhances the glomerular filtration rate and renal plasma flow [8].

He modified and improved a technique that measures the osmolality of samples of proximal tubular fluid of the kidney, which improved the understanding of renal physiology and the reabsorption of specific metabolites, such as glucose and amino acids. In Houston, he created the Renal-Metabolic Laboratory, which he transferred to the San Juan VA Hospital, the first of its kind, founded by a native of the island who brought back knowledge and science capital for improving patient care in Puerto Rico [9].

**Figure 3:**
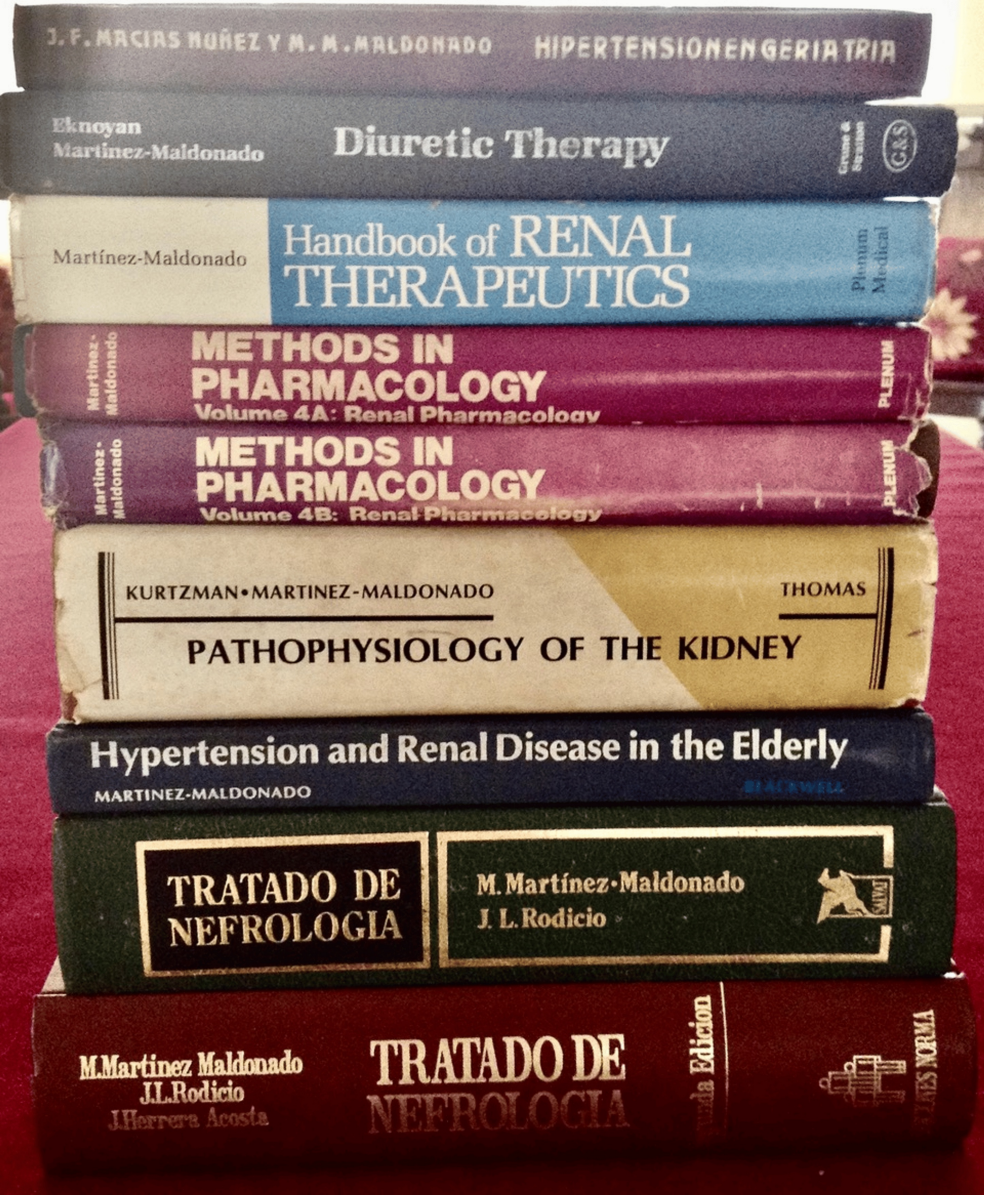
Handbook of Renal Therapeutics and other books edited or co-edited by Dr. Manuel Martinez-Maldonado.

His expertise in internal medicine and focus on nephrology ignited his work in the pathophysiology of heart failure [10], hypertension [11], and the renin-angiotensin system (RAAS) and expanded knowledge regarding the critical role the hormone plays in blood pressure and fluid balance. Because of his efforts to identify specific substrates and molecular pathways within the RAAS, new therapeutic strategies to manage high blood pressure and protect the kidney were developed and documented in his *Handbook of Renal Therapeutics* [12] and other books he edited (Figure 3).

While working at the San Juan VA Medical Center in Puerto Rico, Dr. Martinez-Maldonado began studying electrolyte imbalances in cancer patients. He studied the abnormal states of specific ions and pathological alterations in their plasma concentrations. By analyzing case reports related to electrolyte abnormalities, he concluded that most metastatic diseases leading to adrenal destruction would also result in simultaneous hypoadrenalism and, therefore, show clinical findings of hyponatremia and hyperkalemia [13]. His understanding and recognition of renal physiology intertwined with holistic care exemplifies his scientific mind.

Transition to mentorship and academic leadership

Mentorship

**Figure 4:**
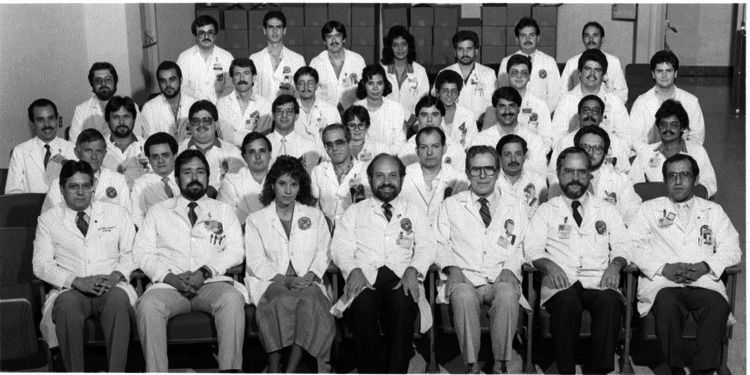
Dr. Manuel Martinez-Maldonado sits in the middle of the front row, surrounded by colleagues and mentees at the San Juan VA Medical Center, Puerto Rico. Dr. Martinez-Maldonado approved the publication of this photograph.

Choosing a treatment and advising one about improving one's health requires scientific knowledge, indefinite inquisitiveness, transparent communication, and constructive compassion. Dr. Martinez-Maldonado epitomizes a mentor willing to take grand initiative despite limiting circumstances. As a role model and determined medical professional, he served as the blueprint for mentees and young Puerto Rican scientists aiming to overcome challenging tasks. In 1973, Dr. Martinez-Maldonado's transition from a dedicated researcher to an influential mentor and academic leader accelerated when he moved back to Puerto Rico as the head of research at the San Juan VA Medical Center. Eighteen months later, he was the San Juan VA Medical Center Chief of Medicine and Chair of the Department of Physiology at the University of Puerto Rico Medical School, teaching medical and doctoral students. From 1973 to 1990, with Dr. Martinez-Maldonado's clinical acumen, his teaching skills, and his budding knack for mentorship, he grew the internal medicine residency training program into a force, the quality of training enjoyed by its residents widely regarded as outstanding, as well as the proficiency and expertise displayed by its residents as a result of his guidance and training. He trained 317 residents and 65 fellows, many of whom went to top institutions in the United States, advancing the reputation of minority physicians and scientists among the broader medical community (Figure 4).

Dr. Martinez-Maldonado's mentorship philosophy is grounded in a rigorous selection of mentees, ensuring that those he guided were capable and committed to advancing in their fields. He emphasized the importance of surrounding oneself with enthusiastic, productive, and creative individuals, fostering an environment of continuous learning and improvement. He believed choosing mentees appropriately for their training and career development was crucial to their success. Dr. Martinez-Maldonado's relationships with the heads of medicine and physiology nationwide allowed him to place his mentees in optimal environments for their growth strategically. Dr. Martinez-Maldonado engaged his mentees in discussions about their career goals and personal challenges, ensuring their well-rounded professional development.

As a member of prestigious societies like the American Society for Clinical Investigation and the Association of American Physicians, he leveraged his extensive network of colleagues to provide his mentees from Puerto Rico and those in the United States with opportunities that otherwise might have been inaccessible. His holistic approach to mentorship is evident in the success stories of many of his mentees, such as Dr. Julio E. Benabe, nephrologist; Dr. Ángel López-Candales, cardiologist; Dr. William Rodriguez and Dr. Jesse Roman, pneumologists, and two PhDs in Pharmacology, Dr. Emma Fernandez-Repollet and Dr. Estela S. Estapé, all of whom became leading figures in their fields.

Academic Leadership

In 1990, Emory University recruited Dr. Martinez-Maldonado as the vice chairman of the Department of Medicine, and the Atlanta VA Medical Center appointed him as head of internal medicine. At Emory, he continued to recruit and develop talented individuals, significantly impacting the medical community. His commitment to excellence in medical education and his innovative approach to integrating clinical practice with research were characteristic of his leadership. The Renal-Metabolic Laboratory studied the molecular aspects and interactions of the renin-angiotensin system in the kidney [14].

In 1998, Dr. Martinez-Maldonado took on the role of Vice-Provost and Vice-President for Research at the Oregon Health Sciences University (OHSU) in Portland, Oregon. During his tenure, he was pivotal in establishing the Vaccine and Gene Therapy Center at OHSU, contributing significantly to the institution's research capabilities. Afterward, he returned to his native Puerto Rico, and from 2000 to 2006, he served as President and Dean of the Ponce School of Medicine. Under his leadership, the school achieved full accreditation and experienced significant financial improvements, solidifying its position as a reputable medical institution. As a result of his accomplishment, the Puerto Rico Association of Industrialists chose him as Executive of the Year for the Southern Section of Puerto Rico.

In 2007, he transitioned to Executive Vice President for Research at the University of Louisville School of Medicine in Kentucky until his retirement. During his tenure, the establishment and successful operation of the Center for Predictive Medicine, a level-3 biodefense laboratory (one of 13 in the United States) highlighted his continued dedication to advancing medical research and preparedness.

**Figure 5:**
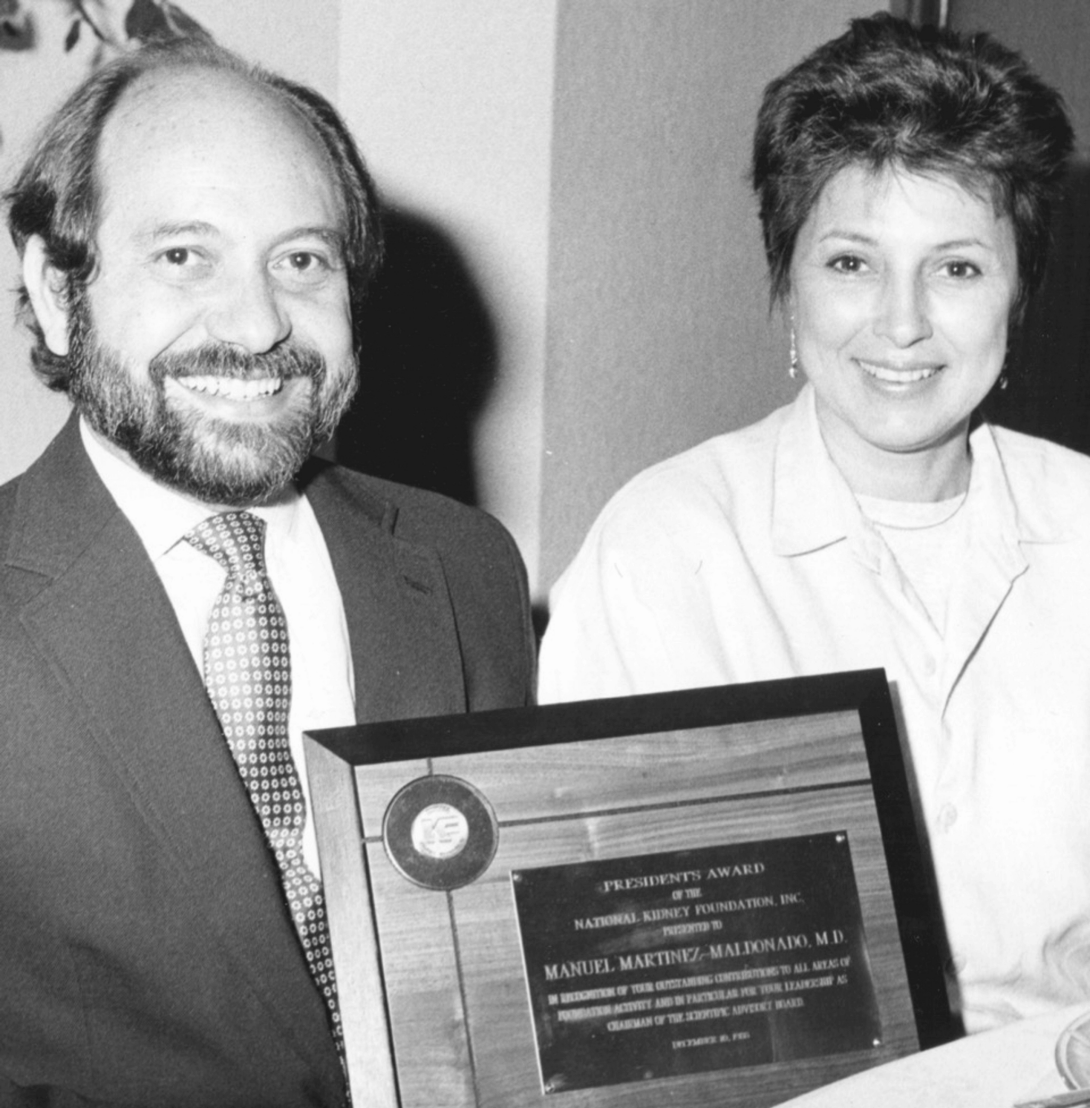
Dr. Manuel Martinez-Maldonado and his wife, Nivia Martinez, with the President's Award of the National Kidney Foundation. Dr. Martinez-Maldonado approved the publication of this photograph.

Dr. Martinez-Maldonado's exemplary contributions to medical science and education have earned him numerous prestigious awards and honors. He received the Southern Society for Clinical Investigation Founder's Medal [15], the President's Award of the National Kidney Foundation, and the Miatello Award from the Latin American Society of Nephrology and Hypertension, among others. He was elected fellow of the American Association for the Advancement of Science, the American Heart Association, and the Council for High Blood Pressure Research. He served in the American Society of Nephrology and was President of the Latin American Society of Nephrology and Hypertension. He has published over 200 articles for various academic journals and served in editorial roles for several publications, including editor-in-chief of the *American Journal of Medical Science*. In 2021, he received the Albert Nelson Marquis Lifetime Achievement Award by Marquis Who's Who [16] for his achievements and leadership qualities (Figure 5).

Current passions

After retiring from medicine in 2009, Dr. Martinez-Maldonado's lifelong passion for literature blossomed. He has published six poetry books and seven novels, one of which is *El Imperialista Ausente* (*The Absent Imperialist*), which won the National Novel Award from the Institute of Puerto Rican Culture in 2013 [17]. Since the 1970s, he has also worked as a film critic and editorial contributor, publishing over 900 movie reviews and writing 120 health-related articles in newspapers and media outlets on the island. He also served as the President of the Board of Directors of the Institute of Puerto Rican Culture and the Center for the Performing Arts of Puerto Rico. 

## Conclusions

Dr. Manuel Martinez-Maldonado's leadership has helped increase funding for kidney research and raised awareness about kidney health. As the President of the Latin American Society of Nephrology and Hypertension, he created a platform for researchers, clinicians, and policymakers to collaborate and address critical issues in nephrology. His advocacy work advanced the nephrology field and highlighted the importance of addressing health disparities and ensuring equitable access to healthcare. Dr. Martinez-Maldonado is also a builder of medical institutions, from the VA (Veterans Affairs) medical centers in Houston, Texas, and San Juan, Puerto Rico; from helping the Ponce School of Medicine obtain full accreditation; to helping create the Center for Preventative Medicine at the University of Louisville. Dr. Martinez-Maldonado has impacted the medical field through a multipronged approach that includes research, mentorship, and institutional foundational creation. A physician by profession, a writer at heart, and a peerless mentor in his soul, he exemplifies the profound impact a dedicated scientist and educator can have on human health.
